# Lymphatic flow mapping using near-infrared fluorescence imaging with indocyanine green helps to predict lymph node metastasis intraoperatively in patients with esophageal or esophagogastric junction cancer not treated with neoadjuvant chemotherapy

**DOI:** 10.1007/s00464-023-10368-4

**Published:** 2023-08-31

**Authors:** Shinichiro Shiomi, Koichi Yagi, Ryohei Iwata, Shoh Yajima, Yasuhiro Okumura, Susumu Aikou, Hiroharu Yamashita, Sachiyo Nomura, Yasuyuki Seto

**Affiliations:** 1 Department of Gastrointestinal Surgery, Graduate School of Medicine, University of Tokyo, 7-3-1 Hongo, Bunkyo-ku, Tokyo, 113-8655 Japan; 2 Division of Digestive Surgery, Department of Surgery, Nihon University School of Medicine, Tokyo, Japan; 3 Department of Surgery, The Institute of Medical Science, The University of Tokyo, Tokyo, Japan

**Keywords:** Esophageal cancer, Indocyanine green, Lymph node metastasis, Near-infrared imaging, Neoadjuvant chemotherapy

## Abstract

**Background:**

Lymphatic flow mapping using near-infrared fluorescence (NIR) imaging with indocyanine green (ICG) has been used for the intraoperative prediction of lymph node metastasis in esophageal or esophagogastric junction cancer. However, a consistent method that yields sufficient diagnostic quality is yet to be confirmed. This study explored the diagnostic utility of our newly established lymphatic flow mapping protocol for predicting lymph node metastasis in patients with esophageal or esophagogastric junction cancer.

**Methods:**

We injected 0.5 mL of ICG (500 μg/mL) into the submucosal layer at four peritumoral points on the day before surgery for 54 patients. We performed lymphatic flow mapping intraoperatively using NIR imaging. After determining the NIR status and presence of metastases, evaluable lymph node stations on in vivo imaging and all resected lymph nodes were divided into four categories: ICG+meta+ (true positive), ICG+meta− (false positive), ICG−meta+ (false negative), and ICG−meta− (true negative).

**Results:**

The distribution of ICG+ and meta+ lymph node stations differed according to the primary tumor site. Sensitivity and specificity for predicting meta+ lymph nodes among ICG+ ones were 50% (95% CI 41–59%) and 75% (73–76%), respectively. Predicting meta+ lymph node stations among ICG+ stations improved these values to 66% (54–77%) and 77% (74–79%), respectively. Undergoing neoadjuvant chemotherapy was an independent risk factor for having meta+ lymph nodes with false-negative diagnoses (odds ratio 4.82; 95% CI 1.28–18.19). The sensitivity of our technique for predicting meta+ lymph nodes and meta+ lymph node stations in patients who did not undergo neoadjuvant chemotherapy was 79% (63–90%) and 83% (61–94%), respectively.

**Conclusion:**

Our protocol potentially helps to predict lymph node metastasis intraoperatively in patients with esophageal or esophagogastric junction cancer undergoing esophagectomy who did not undergo neoadjuvant chemotherapy.

**Graphical abstract:**

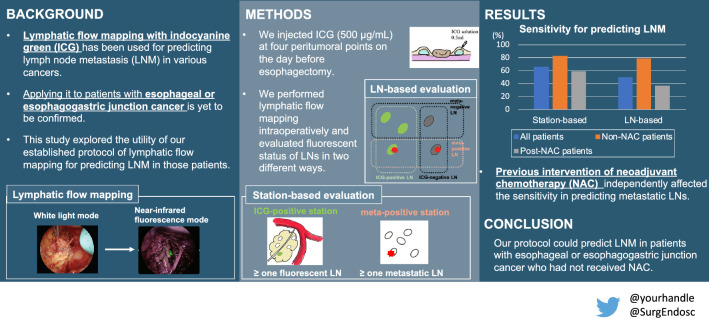

Esophageal cancer has a poor prognosis, even though various treatment strategies have been established [1]. The presence of lymph node metastasis (LNM) is a critical prognostic factor for esophageal cancer [2], making the accurate prediction of metastasis crucial for planning an appropriate treatment course. Preoperatively, LNM is most commonly predicted using the size of lymph nodes (LNs), determined using computed tomography. However, computed tomography has a poor ability to detect LNMs. The most recent edition of the Japanese Classification of Esophageal Cancer states that—using cutoff values of 6 to 8 mm for the nodal diameter—the sensitivity of computed tomography for detecting LNM in patients with cT2-4 esophageal cancer ranges from 27.8 to 65.2% for mediastinal LNs and 52.3% to 81.8% for abdominal LNs [3]. Almost one-third of metastatic LNs reportedly have diameters of less than 5 mm [4]; thus, the features of LNs visualized using computed tomography may provide limited information for accurately predicting LNM in esophageal cancer.

Indocyanine green (ICG), when combined with proteins in the lymphatic fluid, emits light under 760 nm illumination and produces fluorescence at 830 nm [5]. Mapping the lymphatic flow from a tumor using near-infrared fluorescence (NIR) imaging with ICG has been applied to predict LNM intraoperatively in various cancers [6]. Studies have previously explored the feasibility and diagnostic ability of this technique to predict LNM in esophageal and esophagogastric junction cancers [7–15]. A systematic review reported that NIR imaging enabled 89% of sentinel LNs to be identified, and that pooled sensitivity to detect patients with LNM was 84% [8]. However, the concentration of the ICG solution and the latency between injecting it and evaluating fluorescent lymphatic flow differed among the included studies [9–14]. Moreover, esophageal cancer causes a multidirectional occurrence of LNM [16, 17]; therefore, the possibility of developing LNMs from lymphatic pathways other than the sentinel LNs should also be considered. However, an optimal protocol for injecting ICG and evaluating the NIR imaging status to predict sites harboring LNMs has not yet been established.

We previously reported that an endoscopic submucosal injection of 0.5 mL of ICG diluted to 500 μg/mL at four peritumoral points on the day before esophagectomy appeared to be an optimal setting for patients with a single primary tumor. This protocol yielded a sensitivity of 91% for detecting stations with metastatic LNs among the stations with fluorescent LNs [18]. However, this was only a preliminary study with a small number of enrolled patients. The present study aimed to confirm the diagnostic utility of our established protocol for predicting LNM in esophageal and esophagogastric junction cancer and to identify the patient characteristics that support the reliable application of this protocol.

## Materials and methods

### Patients

This prospective, single-institution, interventional study was conducted at the University of Tokyo Hospital. Patients who underwent curative esophagectomy with radical LN dissection for esophageal or esophagogastric junction cancer between October 2020 and December 2022 were enrolled. Each patient was diagnosed according to the 8th edition of the American Joint Committee on Cancer/International Union Against Cancer (AJCC/UICC) staging manual [19] using upper endoscopy with biopsy and computed tomography. We evaluated LNs with a short diameter of ≥ 8 mm on computed tomography as clinically positive for metastasis. The inclusion criteria were as follows: (1) patients with cT1-4aN0-3M0-1 (supraclavicular LN involvement) esophageal or esophagogastric junction cancer; (2) patients with good primary organ function and no requirement for any surgical intervention; and (3) patients who provided written consent after sufficient explanation for participation in this study. The exclusion criteria were as follows: (1) patients with a history of hypersensitivity to iodine-containing drugs; (2) patients diagnosed preoperatively with multiple primary cancers; (3) patients with concurrent head and neck cancer, gastric cancer, or lung cancer, or who had a history of these cancers within the past 5 years; and (4) patients with stenosis due to a tumor or other causes that prevented the injection of ICG on the distal side of the tumor. The protocol was approved by the Ethics Committee of the Faculty of Medicine at the University of Tokyo (approval number 2020003P), and written informed consent was obtained from all participants. This study was conducted in accordance with the Declaration of Helsinki and registered with the UMIN Clinical Trials Registry (Registration number UMIN000042199).

### Preoperative ICG injection technique

**Fig. 1:**
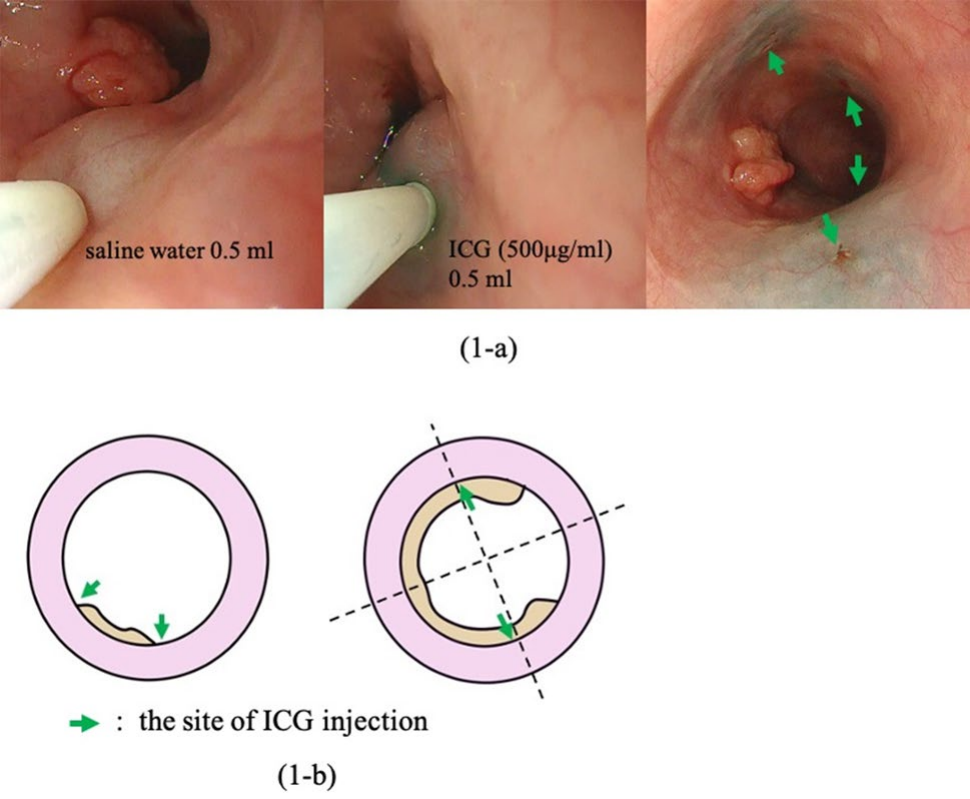
Endoscopic injection of the diluted indocyanine green (ICG) solution. **a** The solution was injected into the submucosal layer of the square around the tumor after saline water was injected. **b** We determined the sites of ICG injection according to whether the tumor was more or less than half the circumference of the esophagus. Green arrows indicate the sites of injection (Color figure online)

Endoscopic submucosal injection of the ICG solution (500 μg/mL) was performed the day before esophagectomy, as previously described [18]. For patients who underwent endoscopic submucosal resection or neoadjuvant chemotherapy, we injected the ICG solution around the scar or remnant lesion, following the location of the tumors, before the primary treatments. To avoid unintended ICG injection into the muscular layer, we first injected 0.5 mL of saline water into each site to create a bleb. Subsequently, 0.5 mL of diluted ICG solution was injected into the bleb (Fig. 1a). For tumors smaller than half the circumference of the esophagus, diluted ICG solution was injected into four quadrants making up a square around the proximal and distal edges of the tumor. For tumors larger than half the circumference of the esophagus, the ICG solution was injecting locally into opposite sides of the lateral walls across the center of the tumor at the proximal and distal sites (Fig. 1b).

### Perioperative treatments and surgical procedures

We determined the treatment strategy according to the 4th and 5th editions of the Esophageal Cancer Practice Guidelines promulgated by the Japan Esophageal Society during the study period [20, 21]. Patients with ≥ cT3 and/or ≥ cN1 disease underwent neoadjuvant chemotherapy followed by curative esophagectomy: three cycles with docetaxel, cisplatin, and 5-fluorouracil (the DCF combination) or three cycles with oxaliplatin and capecitabine (the XELOX combination).

Our established transmediastinal esophagectomy was most frequently performed during the study period, using a combination of a video-assisted transcervical procedure and a transhiatal approach with or without the da Vinci Xi robotic surgical system (Intuitive Surgical, Inc., California, USA). Whereas conventional transthoracic esophagectomy was only performed in some patients. We previously reported that, as for conventional transthoracic esophagectomy, our established transmediastinal esophagectomy allows mediastinal LNs to be resected [22]. Supraclavicular LN dissection was performed in patients with advanced thoracic esophageal cancers who were 75 years or younger. Most reconstructions were performed using a gastric conduit. When the stomach was unavailable, a free jejunal graft was used.

### Intraoperative NIR imaging and pathological evaluation

**Fig. 2:**
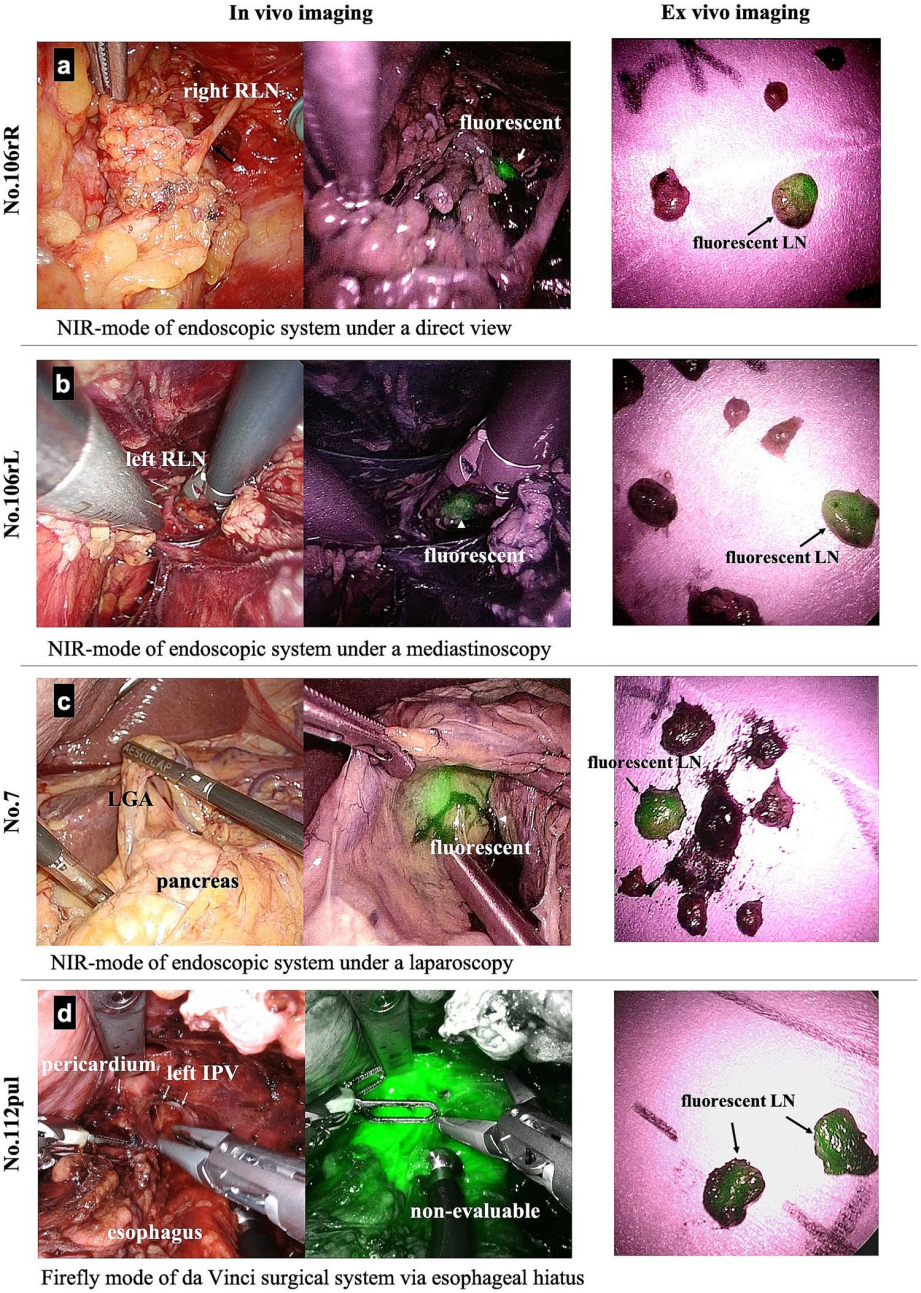
Examples of intraoperative in vivo and ex vivo imaging. **a**–**c** Fluorescence was detected intraoperatively in No. 7, No. 106recR, and No. 106recL. **d** The fluorescence of LNs included in No. 112pul was considered non-evaluable on in vivo imaging due to the overflow of ICG solution. *IPV* inferior pulmonary vein, *LN* lymph node, *NIR* near-infrared fluorescence, *RLN* recurrent laryngeal node

We used the endoscopic fluorescence imaging system VISERA ELITE II (Olympus Co., Ltd., Tokyo, Japan) and the firefly mode of the robotic surgical system for NIR imaging using ICG. We obtained clear real-time images of ICG fluorescence by frequently switching the wavelength between the white-light and NIR imaging modes. We dissected each LN station using the Japanese Classification of Esophageal Cancer [3]. While dissecting the stations, we conducted intraoperative NIR imaging (in vivo imaging) and examined whether each targeted LN station contained fluorescent LNs (Fig. 2a–c). We evaluated the presence or absence of fluorescent LNs in each LN station by moving the lens to a distance of 4–5 cm from the target tissues. Similar to other reports, the presence of fluorescence on NIR imaging was determined through a consensus of more than three surgeons specializing in upper gastrointestinal cancer, as quantitative evaluation of fluorescence intensity was not available through the imaging systems [11, 14]. The LN fluorescence in some stations near the site of ICG solution injection could not be evaluated due to the overflow of the solution into the surrounding non-lymphoid tissues; these stations were defined as “non-evaluable” (Fig. 2d).

After resecting the specimens, we immediately harvested and classified all the LNs into applicable LN stations. Next, we performed direct NIR imaging of each separate LN (ex vivo imaging). We extracted fluorescent LNs from each station and separated them from the LNs without fluorescence, simultaneously submitting them for pathological examination. Pathologists performed histological examination of the primary cancer and examined the presence of metastatic LNs in each station divided by the presence or absence of fluorescence on ex vivo imaging.

### Evaluation of NIR imaging status and metastasis in each LN station

We evaluated the ability of in vivo and ex vivo NIR imaging to predict LNM using LN- and station-based methods. The LN-based evaluation described the fluorescence status using ex vivo imaging and the presence of metastasis using a pathological examination for each LN as “ICG-positive/negative LNs” and “meta-positive/negative LNs,” respectively. The station-based evaluation defined LN stations identified as having one or more fluorescent LNs using intraoperative in vivo imaging as “ICG positive.” LN stations that were identified with no fluorescent LNs on in vivo imaging were defined as “ICG negative.” LN stations that were non-evaluable on in vivo imaging were excluded from the elements targeted for the station-based evaluation. Similarly, LN stations with one or more metastatic LNs were described as “meta positive.” Hence, all resected LNs and evaluable LN stations were classified into four groups according to their NIR imaging status and the presence of metastasis: “ICG positive and meta positive (ICG+meta+ , true positive),” “ICG negative and meta positive (ICG−meta+, false negative),” “ICG positive and meta negative (ICG+meta−, false positive),” and “ICG negative and meta negative (ICG−meta−, true negative).”

### Statistical analyses

We calculated the sensitivity, specificity, positive likelihood ratio (PLR), and negative likelihood ratio (NLR) of the intraoperative NIR fluorescence status to detect meta-positive LNs and LN stations using standard formulae. We determined our sample size based on the sensitivity of detecting LNM using a station-based evaluation: the rate of ICG-positive LN stations among meta-positive LN stations. Using the calculation previously reported for the sample size [23], given a total width of the confidence interval of 0.2, an expected proportion of 0.8–0.9, and a confidence level of 0.05, we estimated that 35–61 meta-positive LN stations were needed. Given that one meta-positive LN station per patient would be obtained, we selected a cohort of 50–70 patients with esophageal cancer or esophagogastric junction cancer as the sample size.

Logistic regression models were used to investigate the characteristics of patients with LNs or LN stations associated with high false-negative rates. Pearson’s chi-square test or Fisher’s exact test was used in univariate analysis, and potentially associated factors were extracted for multivariate analysis. Continuous variables are expressed as median and interquartile range. Statistical significance was set at *P* < 0.05. JMP® 15 (SAS Institute Inc., Cary, NC, USA) was used for all statistical analyses.

## Results

### Patient characteristics

Sixty-two patients, including 60 undergoing transmediastinal esophagectomy and two undergoing transthoracic esophagectomy, agreed to participate in the study and underwent preoperative injection of ICG solution; no adverse events related to the injections were observed. Among the 62 patients, ICG injection could not be completed in one patient due to tumor-derived stenosis, the esophagectomy was interrupted in one patient due to massive tumor invasion, ex vivo imaging data were missing for one patient due to difficulties experienced with the endoscopic fluorescence imaging system, and five patients were diagnosed with multiple esophageal cancers in the final histological examinations; therefore, these patients were excluded from the population for analyses. Thus, data for 54 patients were analyzed in this study. The clinical characteristics of the patients are summarized in Table 1. Most patients, except for one undergoing conventional transthoracic esophagectomy, underwent our established transmediastinal esophagectomy. The median number of resected LN stations and LNs per patient were 21 (21–22) and 54 (44.5–67), respectively. Thirty patients were diagnosed with having LNM.

**Table 1:** Clinical features and short-term surgical outcomes of the enrolled patient group

Feature/outcome	Total (*n* = 54)
Age, years (median [IQR])	69 [61–74]
Sex (*n*)	
Male/female	48/6
Body mass index, kg/m^2^ (median [IQR])	22.0 [19.9–24.4]
Preoperative treatment (*n*)	
NAC/ESD/none	26/6/22
Neoadjuvant chemotherapy (*n*)	
DCF/XELOX	25/1
Primary tumor location (*n*)	
Ce/Ut/Mt/Lt/Jz	1/5/14/21/13
Tumor length, cm (median [IQR])	4.0 [2.8–5.5]
Tumor circumference, % (median [IQR])	67 [40–90]
Endoscopic appearance of the primary tumor (*n*)	
0-I/0-IIa/0-IIb/0-IIc/0-III	2/6/3/8/1
1/2/3/4	4/18/11/1
Tumor infiltration depth (cT) (*n*)	
cT1/cT2/cT3/cT4	18/8/25/3
Nodal involvement (cN) (*n*)	
cN0/cN1/cN2/N3	28/20/4/2
cStage (UICC 8th) (*n*)	
I/II/III/IV	17/11/20/6
Tumor infiltration depth after NAC (CT-T) (*n*)	
CT-T0/CT-T1/CT-T2/CT-T3/CT-T4	1/5/7/12/1
Nodal involvement after NAC (CT-N) (*n*)	
CT-N0/CT-N1/CT-N2/CT-N3	13/12/0/1
Esophagectomy approach (*n*)	
Transthoracic/transmediastinal	1/53
Lymph-node dissection (*n*)	
3-Field/2-field	30/24
Operative time, min (median [IQR])	424 [374–466]
Complication rates (CD classification) (*n*)	
≤ 1/2/3a/ ≥ 3b	27/12/12/3
Histological classification (*n*)	
Squamous cell carcinoma	38
Basaloid cell carcinoma	1
Adenocarcinoma	13
Adenosquamous cell carcinoma	1
Neuroendocrine carcinoma	1
Tumor infiltration depth (pT) (*n*)	
pT0/pT1/pT2/pT3/pT4	3/22/8/18/3
Nodal involvement (pN) (*n*)	
pN0/pN1/pN2/pN3	24/14/10/6

*CD* Clavien–Dindo, *Ce* cervical esophagus, *DCF* docetaxel, cisplatin, and 5-fluorouracil combination, *ESD* endoscopic submucosal dissection, *IQR* interquartile range, *Jz* junctional zone, *Lt* lower thoracic esophagus, *Mt* middle thoracic esophagus, *NAC* neoadjuvant chemotherapy, *UICC 8th* the eighth edition of the UICC TNM classification, *Ut* upper thoracic esophagus, *XELOX* oxaliplatin and capecitabine combination

### Classification of LNs or LN stations according to NIR fluorescence status and the presence of metastasis

We resected 2989 LNs, comprising 1152 LN stations. A total of 240 LN stations were non-evaluable using in vivo imaging. The distribution of groups categorized according to the NIR fluorescence status and histological diagnoses is summarized in Table 2.

**Table 2:** Classification of resected LNs and LN stations according to near-infrared fluorescence imaging status and the presence of lymph node metastases

Resected LNs, *n*	ICG+	ICG−	Total
Meta+	55	55	110
Meta−	723	2156	2879
Total	778	2211	2989

Resected LN stations, *n*	Evaluable		Non-evaluable	Total
	ICG+	ICG−		
Meta+	41	21	15	77
Meta−	198	652	225	1075
Total	239	673	240	1152

*Meta* metastasis, *ICG* indocyanine green, *LN* lymph node

**Fig. 3:**
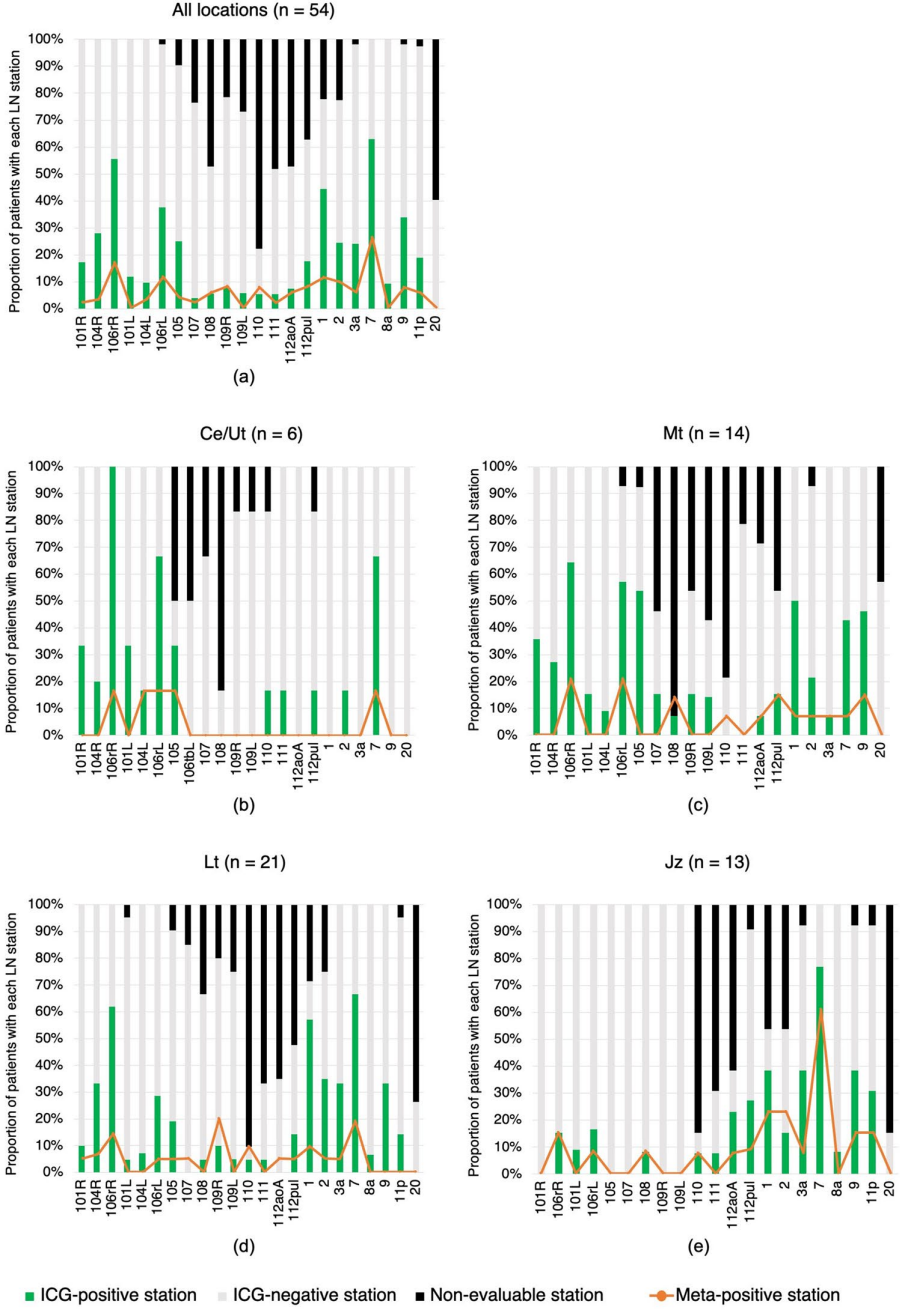
Proportion of patients with each type of near-infrared fluorescence status on in vivo imaging and pathological status of each dissected LN station for the entire patient group (**a**) and subgroups with tumor locations of Ce or Ut (**b**), Mt (**c**), Lt (**d**), and Jz (**e**). LN stations examined for a few patients were excluded from the horizontal axis lists. *Ce* cervical esophagus, *Jz* junctional zone, *LN* lymph node, *Lt* lower thoracic esophagus, *Mt* middle thoracic esophagus, *Ut* upper thoracic esophagus

Figure 3 shows the proportion of ICG-positive and meta-positive results at each LN station for the entire patient group and subgroups classified according to tumor site. LN stations with a high proportion of ICG-positive results showed a high frequency of the presence of LNM. LNs along the right and left recurrent laryngeal nerves (No. 106recR and No. 106recL), right cardiac LNs (No. 1), and LNs along the left gastric artery (No. 7) were included in the top four LN stations with the highest rates of ICG-positive and meta-positive results (Fig. 3a). Moreover, the distribution of ICG-positive LN stations differed according to the primary tumor site. Patients with cancer located lower down the esophagus had a low rate of ICG-positive results at the cervical or upper-mediastinal LN stations and a high rate of ICG-positive results at the abdominal LN stations (Fig. 3b–e).

### Risk factors associated with a false-negative diagnosis of LNM using NIR imaging

As shown in Table 2, 55 of the 110 resected meta-positive LNs were evaluated as ICG negative on ex vivo imaging. Among the evaluable LN stations, 21 of the 62 meta-positive LN stations were evaluated as ICG negative on in vivo imaging. Thus, they were described as false-negative results. Table 3 shows the results of the univariate and multivariate analyses conducted to assess the risk factors for patients with a high rate of false-negative diagnoses observed on the LN-based evaluation and station-based examination. In the univariate analysis, a tumor length of ≥ 5 cm, tumor infiltration depth of cT3 or deeper, tumor circumference of ≥ 50%, and being treated with neoadjuvant chemotherapy were identified as potential risk factors for false-negative diagnoses on the LN-based examination. The multivariate analysis incorporating these four factors revealed that being treated with neoadjuvant chemotherapy (odds ratio [OR] 4.82; 95% confidence interval [CI] 1.28–18.19; *P* < 0.05) was an independent risk factor for having LNs with false-negative results. Neoadjuvant chemotherapy was also associated with a high rate of false-negative diagnoses in station-based evaluations. However, the difference was insignificant both in the univariate (OR 3.46; 95% CI 0.87–13.73; *P* = 0.07) and multivariate (OR 2.96; 95% CI 0.72–12.18; *P* = 0.13) analyses.

**Table 3:** Risk factors of patients having meta-positive LNs and LN stations associated with false-negative results

	ICG+ LNs, *n* (%)	ICG− LNs, *n* (%)	Univariate analysis		Multivariate analysis	
			Odds ratio (95% CI)	*P* value	Odds ratio (95% CI)	*P* value
Neoadjuvant chemotherapy						
Performed	28 (37%)	48 (63%)	6.61 (2.55–17.15)	< 0.01	4.82 (1.28–18.19)	< 0.05
Not performed	27 (79%)	7 (21%)	1		1	
Main tumor location						
EC (Ce/Ut/Mt/Lt)	30 (46%)	35 (54%)	1.46 (0.68–3.13)	0.33		
EGJC (Jz)	25 (56%)	20 (44%)	1			
Tumor infiltration depth (cT)						
≥ cT3	41 (44%)	52 (56%)	5.92 (1.59–21.99)	< 0.01	1.38 (0.27–7.18)	0.70
≤ cT2	14 (82%)	3 (18%)	1		1	
Nodal involvement (cN)						
≥ N1	42 (47%)	47 (53%)	1.82 (0.69–4.82)	0.22		
N0	13 (62%)	8 (38%)	1			
Tumor length						
≥ 5 cm	32 (43%)	43 (57%)	2.58 (1.12–5.93)	< 0.05	1.93 (0.76–4.91)	0.17
< 5 cm	23 (66%)	12 (34%)	1		1	
Tumor circumference						
≥ 50%	45 (46%)	52 (54%)	3.85 (1.00–14.87)	< 0.05	1.26 (0.25–6.40)	0.78
< 50%	10 (77%)	3 (23%)	1		1	
Endoscopic appearance of the tumor						
Depressed lesion	43 (50%)	43 (50%)	1.00 (0.40–2.47)	1.00		
Other	12 (50%)	12 (50%)	1			
Histological classification						
Squamous cell	29 (54%)	25 (46%)	0.75 (0.35–1.58)	0.45		
Other	26 (46%)	30 (54%)	1			

	ICG+ LN stations, *n* (%)	ICG−LN stations, *n* (%)	Univariate analysis		Multivariate analysis	
			Odds ratio (95% CI)	*P* value	Odds ratio (95% CI)	*P* value
Neoadjuvant chemotherapy						
Performed	26 (59%)	18 (41%)	3.46 (0.87–13.73)	0.07	2.96 (0.72–12.08)	0.13
Not performed	15 (83%)	3 (17%)	1		1	
Main tumor location						
EC (Ce/Ut/Mt/Lt)	27 (71%)	11 (29%)	1.33 (0.20–1.67)	0.30		
EGJC (Jz)	14 (58%)	10 (42%)	1			
Tumor infiltration depth (cT)						
≥ T3	32 (63%)	19 (37%)	2.67 (0.52–2.67)	0.23		
≤ T2	9 (82%)	2 (18%)	1			
Nodal involvement (cN)						
≥ N1	34 (65%)	18 (35%)	1.24 (0.28–5.36)	0.78		
N0	7 (70%)	3 (30%)	1			
Tumor length						
≥ 5 cm	23 (59%)	16 (41%)	2.50 (0.77–7.76)	0.12	2.07 (0.61–6.98)	0.24
< 5 cm	18 (78%)	5 (22%)	1		1	
Tumor circumference						
≥ 0.5	36 (65%)	19 (35%)	1.32 (0.23–7. 46)	0.75		
< 0.5	5 (71%)	2 (29%)	1			
Endoscopic appearance of a tumor						
Depressed lesion	32 (64%)	18 (36%)	1.69 (0.59–7.04)	0.47		
Other	9 (75%)	3 (25%)	1			
Histological classification						
Squamous cell	23 (74%)	8 (26%)	0.48 (0.16–1.41)	0.18		
Other	18 (58%)	13 (42%)	1			

*Ce* cervical esophagus, *CI* confidence interval, *EC* esophageal cancer, *EGJC* esophagogastric junction carcinoma, *ICG−* indocyanine green negative, *ICG+* indocyanine green positive, *Jz* junctional zone, *Lt* lower thoracic esophagus, *Mt* middle thoracic esophagus, *Ut* upper thoracic esophagus

### Diagnostic quality of NIR imaging for detecting LNM in each evaluation method

The sensitivity, specificity, PLR, and NLR for detecting LNM obtained through LN-based evaluation were 50% (95% CI 41–59%), 75% (73–76%), 2.0 (1.6–2.4), and 0.67 (0.55–0.81), respectively. The respective values by station-based evaluation were 66% (54–77%), 77% (74–79%), 2.8 (2.3–3.5), and 0.44 (0.31–0.63). Table 4 summarizes the diagnostic values of each evaluation method for predicting LNM in patients who did not undergo neoadjuvant chemotherapy and in those who did. Patients who did not undergo neoadjuvant chemotherapy had better sensitivity, PLR, and NLR for detecting LNM than patients who underwent this treatment, regardless of the evaluation method used.

**Table 4:** Diagnostic values for predicting lymph node metastasis based on different methods of evaluation

All patients (*n* = 54)	Sensitivity (95% CI), TP/TP + FN	Specificity (95% CI), TN/FP + TN	PLR (95% CI)	NLR (95% CI)
LN-based evaluation	50 (41–59)	75 (73–76)	2.0 (1.6–2.4)	0.67 (0.55–0.81)
	55/110	2156/2879		
Station-based evaluation	66 (54–77)	77 (74–79)	2.8 (2.3–3.5)	0.44 (0.31–0.63)
	41/62	652/850		

	Sensitivity (95% CI)	Specificity (95% CI)	PLR (95% CI)	NLR (95% CI)
Non-NAC patients (*n* = 28)				
LN-based evaluation	79 (63–90)	71 (69–74)	2.8 (2.3–3.3)	0.29 (0.15–0.56)
	27/34	1037/1454		
Station-based evaluation	83 (61–94)	76 (72–80)	3.5 (2.7–4.6)	0.22 (0.08–0.61)
	15/18	343/449		
Post-NAC patients (*n* = 26)				
LN-based evaluation	37 (27–48)	79 (76–81)	1.7 (1.3–2.3)	0.80 (0.68–0.96)
	28/76	1118/1424		
Station-based evaluation	59 (44–72)	77 (73–81)	2.6 (1.9–3.5)	0.53 (0.37–0.76)
	26/44	309/401		

*CI* confidence interval, *FN* false negative, *FP* false positive, *LN* lymph node, *NAC* neoadjuvant chemotherapy, *NLR* negative likelihood ratio, *PLR* positive likelihood ratio, *TN* true negative, *TP* true positive

Additionally, of the 18 meta-positive LN stations evaluated in patients who did not undergo neoadjuvant chemotherapy, only five were diagnosed to be metastatic through preoperative computed tomography using cutoff values of 8 mm for the nodal diameter. Thus, in this patient group, our technique could predict meta-positive LN stations with greater sensitivity compared to preoperative computed tomography (83% [61–94%] vs. 28% [13–51%]).

### Diagnostic quality of our technique for predicting LNM according to anatomical areas

**Fig. 4:**
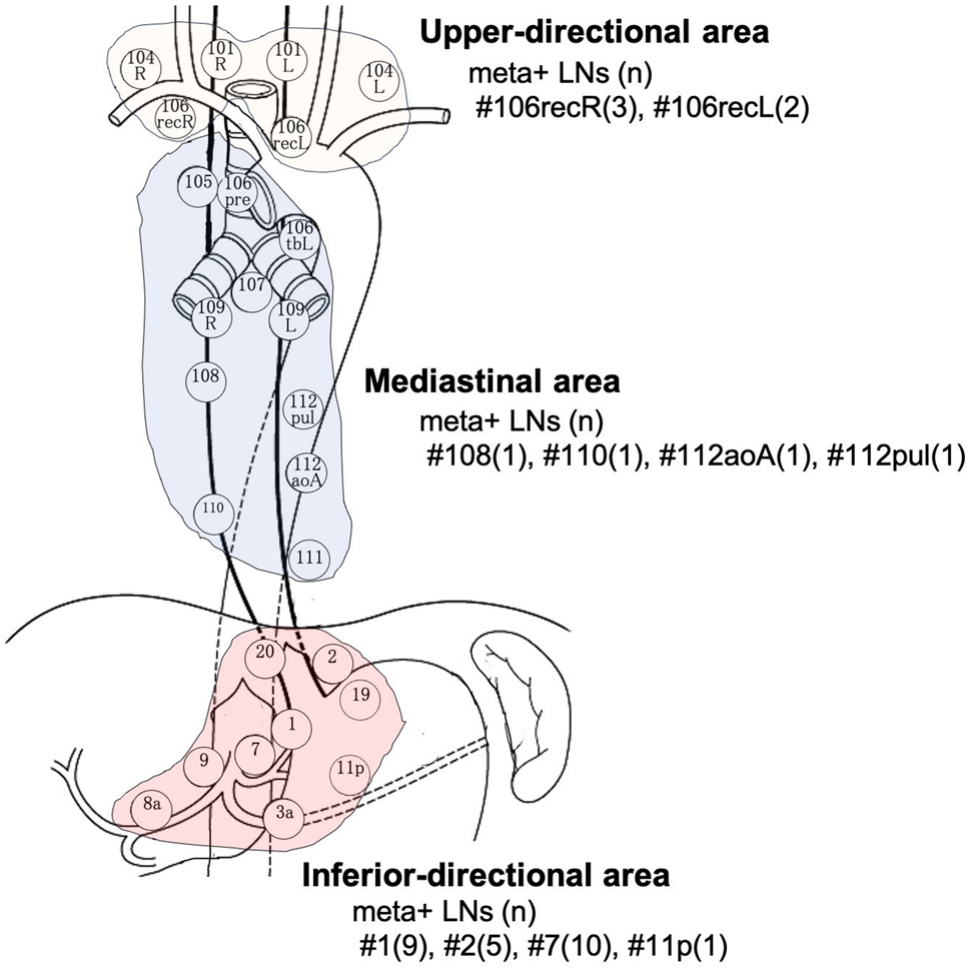
Classification of LN stations according to the anatomical areas and the number of meta-positive LNs in LN stations consisting of each group

To examine the difference in diagnostic values among anatomical areas, we classified all LNs and LN stations into three areas according to the directions of lymphatic flow (Fig. 4). Diagnostic values by each evaluation method in each area for patients who did not receive neoadjuvant chemotherapy are presented in Table 5. LNs in the upper-directional area, including recurrent laryngeal nerve LNs and supraclavicular LNs, had better PLR and NLR for predicting LNM using LN-based evaluation than the other areas. Furthermore, all meta-positive LN stations included in the upper-directional area could be detected via station-based evaluation.

**Table 5:** Diagnostic values in each anatomical area divided by the directions of lymphatic flow for patients who underwent upfront surgery

	ICG+meta+, *n*	ICG−meta+, *n*	ICG+meta−, *n*	ICG−meta−, *n*	Sensitivity, %	Specificity, %	PLR	NLR
LN-based evaluation								
Upper-directional area	4	1	78	347	80	82	4.4	0.25
Mediastinal area	2	2	102	306	50	75	2.0	0.67
Inferior-directional area	21	4	237	384	84	62	2.2	0.26
Station-based evaluation								
Upper-directional area	5	0	39	94	100	71	3.4	0.00
Mediastinal area	1	1	18	139	50	89	4.4	0.57
Inferior-directional area	9	2	49	110	82	69	2.7	0.26

*NLR* negative likelihood ratio, *PLR* positive likelihood ratio

## Discussion

The present study revealed that our established protocol, which visualizes lymphatic flow in multiple directions using NIR imaging, allowed for the intraoperative prediction of LNM in patients with esophageal cancer and esophagogastric junction cancer who have not undergone neoadjuvant chemotherapy.

Lymphatic flow mapping using NIR imaging with ICG was initially used to detect sentinel LNs in breast cancer [5]. Sentinel LNs are defined as the first LNs or pathways that receive lymphatic flow from the primary tumor. Therefore, diagnosing the presence of LNM in sentinel LNs helps determine whether it is applicable to reduce the extent of LN dissection or organ resection. This technique is based on the principle that sentinel LNs are the first possible sites for LNM to occur along the route of lymphatic drainage from the tumor. A study that investigated the feasibility of applying this technique to esophageal cancer reported that the intraoperative lymphatic flow mapping obtained by injecting ICG after initiating anesthesia had a sensitivity of 43% and a specificity of 59% for predicting metastatic LNs among fluorescent LNs [7]. These were inadequate as parameters for determining whether surgery could be minimized. Hence, in patients with esophageal cancer, LNs into which the injected ICG solution is initially drained and those into which tumor cells derived from the primary cancer are carried may differ greatly. This discrepancy may be partly due to the complex anatomical constitution of the lymphatic pathways in the thoracic esophagus [24] and differences in the density of submucosal lymphatic vessels among individual patients [25]. Therefore, identifying sentinel LNs using submucosal ICG injection might not be sufficiently effective for predicting sites that potentially harbor LNMs.

The current study showed that our established protocol of lymphatic flow mapping—injecting ICG diluted to 500 μg/mL around the tumor on the morning of the day before esophagectomy—yielded a higher sensitivity of detecting meta-positive LN stations among ICG-positive LN stations than detecting meta-positive LNs among ICG-positive LNs; these results were consistent with those of our previous study [18]. For patients with esophageal cancer or esophagogastric junction cancer, setting a longer latency time between ICG injection and NIR imaging, and using station-based evaluation, may enable us to effectively predict sites where LNMs are developing.

In this study, we found that previous treatment with neoadjuvant chemotherapy was an independent risk factor for false-negative results for meta-positive LNs. Patients with esophageal cancer or esophagogastric cancer who had not undergone neoadjuvant chemotherapy showed good sensitivity and positive and negative likelihood ratios for predicting LNM. However, for patients who received neoadjuvant chemotherapy, our method had limited efficacy for the intraoperative prediction of LNM. This suggests that NIR-guided esophagectomy using our protocol may show reliability only in patients who are scheduled to undergo upfront surgery.

Several possible reasons explaining the decline in sensitivity in detecting metastatic LNs in patients who received neoadjuvant chemotherapy may be proposed. First, as the lymphatic pathway differs in each layer of the esophagus wall, patients with advanced esophageal cancer may initially have a different lymphatic flow carrying tumor cells from patients with superficial cancer [26, 27]. Second, researchers have proposed that neoadjuvant chemotherapy increased a false-negative rate of sentinel LN biopsy in patients with breast cancer with LNM because the lymphatic pathway to metastatic LNs is blocked and an alternative lymphatic route through chemotherapy-induced fibrosis is generated [28–30]. These histological modifications in lymphatic flow may also occur in patients with esophageal cancer or esophagogastric junction cancer who received neoadjuvant chemotherapy. However, which mechanism chiefly affected the diagnostic quality remains unclear because approximately 70% of patients with advanced cancer in this study (25 of 36) received neoadjuvant chemotherapy. Thus, our established protocol of NIR imaging may enable us to predict LNM in each LN station and determine the application for the minimized surgery in patients with early-stage esophageal cancer and esophagogastric junction cancer, who are likely to be appropriate for upfront surgery. However, due to the small sample size when restricted to cases undergoing upfront surgery, we cannot ensure that our method is effective in patients with advanced cancer who did not receive neoadjuvant chemotherapy. A greater accumulation of cases undergoing upfront surgery is required to confirm the patient characteristics that our strategy is best suited to.

In this study, we were frequently unable to determine the NIR fluorescence status of the LNs near the primary tumor intraoperatively, regardless of the surgical approaches we selected. The ICG solution overflowing into the non-lymphoid tissues surrounding the injection site may easily spread over the narrow mediastinal space, making the discrimination of fluorescent LNs from non-lymphatic tissues difficult. Hence, our technique may not predict LNM occurring near the primary tumor. However, these LN stations are usually included in the resected area regardless of the likelihood of them harboring an LNM. Therefore, ascertaining whether LN stations distant from the primary tumor have LNM is critical for determining the appropriateness of minimizing surgical intervention. Early establishment of LNM in distant regions, including LNs along the recurrent laryngeal nerve or perigastric LNs, is reportedly caused by the longitudinal submucosal lymphatic pathway in the esophagus [24, 31]. Our results showed that in vivo NIR imaging is able to visualize the lymphatic flow to these distant regions; this visualization enabled us to diagnose each targeted LN station as ICG positive or ICG negative. Furthermore, in patients who did not receive neoadjuvant chemotherapy, we were able to predict meta-positive LNs and meta-positive stations developing in the LN station No. 106recR and No. 106recL with high diagnostic quality. Thus, our technique may effectively estimate lymphatic flow associated with LNM development along this pathway.

Of note, the LN dissection of the LN stations No. 106recR and No. 106recL is the most critical procedure in any esophagectomy. Dissecting these stations poses a risk of developing postoperative laryngeal nerve paralysis, which leads to pulmonary infections and malnutrition due to dysphagia [32–34]. Therefore, selecting patients in whom omitting the dissection of these LNs is indicated may be necessary. The LNM rates in LNs along the recurrent laryngeal nerve in patients with thoracic esophageal cancer reportedly decrease as primary cancers are located at lower sites. However, even in patients with lower thoracic esophageal cancer, those rates in No. 106recR and No. 106recL LNs are still around 10% [35]. Patients with esophagogastric junction cancer reportedly have an LNM rate in No. 106recR LNs exceeding 5% [36]. Hence, even if the preoperative examination did not show LNM at these stations, omitting them from the dissection would be impractical, given the risk of unresected metastatic LNs. Especially in patients with early-stage cancer such as these, our NIR imaging protocol may effectively serve as a tool to determine the need for dissecting the LN stations along the right and left recurrent laryngeal nerves.

Contrary to this, reducing the extent of lymphadenectomy may not be recommended for patients with advanced cancer because the tumor infiltration depth strongly correlates with a high probability of having pathological metastatic LNs [37]. However, thorough dissection of bilateral recurrent laryngeal LNs may occasionally be too invasive for certain patients, even in advanced cancer cases. In patients who are already scheduled to sacrifice one side of the recurrent laryngeal nerves due to tumor invasion or other reasons, we would need to consider protecting the other side. For such cases, NIR-guided esophagectomy using our protocol may aid in determining the prioritization of LNs to be resected in the stations No. 106recR and No. 106recL according to the fluorescence status of each LN. However, most patients with tumor invasion to recurrent laryngeal nerves may frequently undergo neoadjuvant chemotherapy as per the current esophageal cancer guideline. Therefore, in such cases, NIR-guided surgery using our technique may result in high rates of false-negative diagnoses. Hence, omitting the non-fluorescent LN resection should be indicated only to avoid postoperative recurrent nerve paralysis in exchange for the possibility of remaining metastatic LNs. Furthermore, several recent studies have suggested the association of preoperative sarcopenia with the development of dysphagia after esophagectomy [38, 39]. In patients with advanced cancer with severe sarcopenia, preventing postoperative malnutrition due to dysphagia may have a higher priority than performing the complete LN dissection.

The current study had several limitations. First, LN stations containing thick fat tissues or covered by other organs may be difficult to evaluate the ICG fluorescent status accurately because infrared rays can only reach some LNs in the targeted LN station. Therefore, this technical limitation should be acknowledged and each LN station should be observed considering the possibility of missing fluorescent LNs in targeting stations with rich adipose tissues. Second, this study did not clarify whether omitting LN dissection in ICG-negative LN stations would result in safe surgical outcomes. Further prospective studies are required to investigate the potential oncological benefits of reduced lymphadenectomies.

In conclusion, our protocol of preoperative ICG injection and intraoperative lymphatic flow mapping using NIR imaging effectively enabled us to predict LN stations that potentially have nodal involvement in patients with esophageal or esophagogastric junction cancer who had not received neoadjuvant chemotherapy.
